# Angle-Engineered Bi_0.94_La_0.06_CuSeO Thin Films for High-Performance Transverse Thermoelectric Devices

**DOI:** 10.3390/s25092791

**Published:** 2025-04-29

**Authors:** Mingjing Chen, Chenming Yue, Tianchang Qin, Haixu Liu, Guoying Yan, Shufang Wang

**Affiliations:** 1 Hebei Key Laboratory of Energy Metering and Safety Testing Technology, National & Local Joint Engineering Research Center of Metrology Instrument and System, College of Quality and Technical Supervision, Hebei University, Baoding 071002, China; luckymingjing@163.com (M.C.);; 2 Hebei Key Laboratory of Optic-Electronic Information and Materials, College of Physics Science and Technology, Hebei University, Baoding 071002, China; 3 Engineering Research Center of Zero-Carbon Energy Buildings and Measurement Techniques, Ministry of Education, Baoding 071002, China

**Keywords:** transverse thermoelectric effect, angle-engineered, optical and thermal sensors, inclined BiCuSeO thin films

## Abstract

BiCuSeO has emerged as a highly promising material for transverse thermoelectric (TTE) applications, with its performance significantly enhanced through La doping. In this study, we investigate the effect of inclination angle on the TTE performance of inclined Bi_0.94_La_0.06_CuSeO thin films fabricated using the pulsed laser deposition technique. A huge output voltage of 31.4 V was achieved in the 10° inclined Bi_0.94_La_0.06_CuSeO film under 308 nm ultraviolet pulsed laser irradiation. Furthermore, the films also exhibited significant response with excellent linearity when exposed to continuous-wave lasers across a broad spectral range (360 nm to 10,600 nm) and a point-like heat source. Notably, the voltage is directly proportional to sin2*θ*, where *θ* is the inclination angle. These findings not only provide a clear optimization strategy for TTE performance through inclination angle engineering but also highlight the material’s great potential for developing high-performance optical and thermal sensing TTE devices.

## 1. Introduction

The transverse thermoelectric (TTE) effect, a unique thermoelectric phenomenon arising from Seebeck coefficient anisotropy, is characterized by the perpendicular orientations between the temperature gradient and output voltage [1,2,3,4,5]. When an inclined thin film is irradiated by a light or heat source, a temperature difference (∆*T*) arises between the top and bottom surfaces of the film, generating a transverse voltage signal (*V*_x_). This relationship is described by the following equation [1]:

(1) $$V_{x}=∆T∆S\frac{l}{2d}\sin\left(2\theta \right)$$

where ∆*S* = |*S*_ab_ − *S*_c_| represents the Seebeck coefficient difference between the *ab* plane and the *c*-axis of the materials, *l* is the irradiation length on the film, *d* is the film thickness, and *θ* is the inclination angle. The equation reveals that the output voltage is directly proportional to ∆*T*, ∆*S*, *l*, and sin2*θ*, while inversely proportional to *d*.

Over the past three decades, the TTE effect has garnered increasing attention due to the advantages of high sensitivity, efficient response, broad wavelength detection capability without amplification or cooling, and so on [6,7,8,9,10]. Extensive efforts have focused on the optimization of voltage sensitivity and response time by element doping, controlling the inclination angle or film thickness, and the incorporation of absorption layers within limited TTE materials, like YBa_2_Cu_3_O_7_ (high-temperature superconductor oxides), La_1−x_Ca_x_MnO_3_ (colossal magneto resistance manganese oxides), Bi_2_Sr_2_Co_2_O_y_ (misfit-layered cobalt oxides), BiCuSeO (layered oxyselenides), and so on [11,12,13,14,15,16]. BiCuSeO with a natural superlattice presents a typical layered ZrSiCuAs structure, in which the conductive layers (Cu_2_Se_2_)^2−^ and the insulating layers (Bi_2_O_2_)^2+^ are alternately stacked on the *c*-axis, with significant anisotropy between the layers. Improvements in the voltage sensitivity of BiCuSeO thin films have been achieved through element doping with Pb, Ba, and La [17,18,19,20]. In our previous work, La-doped BiCuSeO demonstrated greatly enhanced voltage sensitivity from 1.77 V/mJ to 15.7 V/mJ under 308 nm pulsed laser irradiation, from 1200 μV/W to 6800 μV/W under 360 nm continuous-wave (CW) laser irradiation, and from 1100 μV cm^2^/W to 1920 μV cm^2^/W under heat source irradiation [20]. The La-doped BiCuSeO TTE detector exhibited excellent ultraviolet (UV) pulsed photodetection, ultrabroad spectral detection ability, as well as thermal detection performance. However, the influence of inclination angle on its photo- and thermal detection performance is still unexplored.

In this study, *c*-axis inclined Bi_0.94_La_0.06_CuSeO thin films with different inclination angles were successfully fabricated by the pulsed laser deposition technique, and their TTE performance was systematically investigated. The output voltage increased with sin2*θ* and exhibited a linear dependence on laser energy density, power density, and heat flux density. Therefore, the TTE performance of Bi_0.94_La_0.06_CuSeO thin film devices can be enhanced by controlling the inclination angle.

## 2. Materials and Methods

A series of *c*-axis inclined Bi_0.94_La_0.06_CuSeO thin films were fabricated on LaAlO_3_ (00l) single-crystal substrates using the pulsed laser deposition technique due to its unique ability to produce thin films bearing the stoichiometry close to that of the ceramic target material. The inclination angles of the thin films were 3°, 5°, and 10°, respectively, which were controlled by changing the tilted angles of the substrate. The thickness of the inclined thin film was 200 nm. The laser energy density was about 1.5 J/cm^2^. The repetition rate was 5 Hz. The distance between the target and the substrate was about 50 mm. The argon pressure was about 0.1 Pa. The substrate temperature was about 330 °C. Detailed procedures for the film deposition and characterization methods can be found in our previous studies [3,17,20]. The crystal structure and surface microstructure were analyzed using an X-ray diffractometer (XRD, Bruker AXS D8 advanced, Karlstruer, Germany) and a field emission scanning electron microscope (SEM, FEI Nova NanoSEM450, Brno, Czech Republic), respectively.

**Figure 1:**
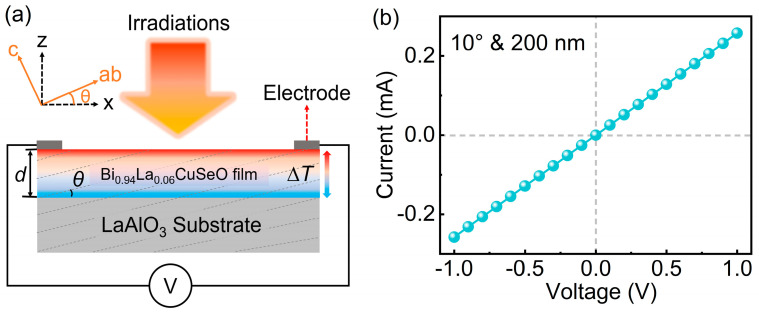
(**a**) Schematic illustration of TTE effect measurement. (**b**) *I*-*V* curve between indium electrodes and inclined Bi_0.94_La_0.06_CuSeO thin film.

For TTE effect measurement, two indium electrodes separated by about 7 mm were deposited symmetrically on the film surface, as illustrated in Figure 1a. The linear current–voltage (*I*-*V*) characteristic shown in Figure 1b confirmed the good ohmic contact between the electrodes and thin film. A 308 nm UV pulsed laser, five CW lasers with wavelengths ranging from UV to far infrared (FIR), and a thermal heater were employed as heating sources. The laser spot size of pulsed laser on the film surface was 5 × 2 mm^2^. The laser energy density (*E*_d_, defined as the ratio of laser energy *E* to irradiation area) ranged from 0.05 to 0.2 mJ/mm^2^. Voltage signals were recorded using a digital oscilloscope with input impedance 1 MΩ (Agilent DSO9254A, Baden-Württemberg, Germany). For CW lasers irradiation, the spot diameter on the film was approximately 1 mm, with power densities ranging from 2 W/cm^2^ to 9 W/cm^2^. For thermal heater irradiation, the distance between the heat source and the film was maintained at 0.5 mm. The heat flux density ranged from 0.25 W/cm^2^ to 1.25 W/cm^2^, calibrated using a commercial Gardon gauge (GD-B3-100K, BEST, Beijing, China). A 2400 Keithley source (Beaverton, OR, USA) meter was used to collect the output voltage signal under both CW lasers and heat source irradiation.

## 3. Results

### 3.1. Thin Film Crystal Structure Characterization

**Figure 2:**
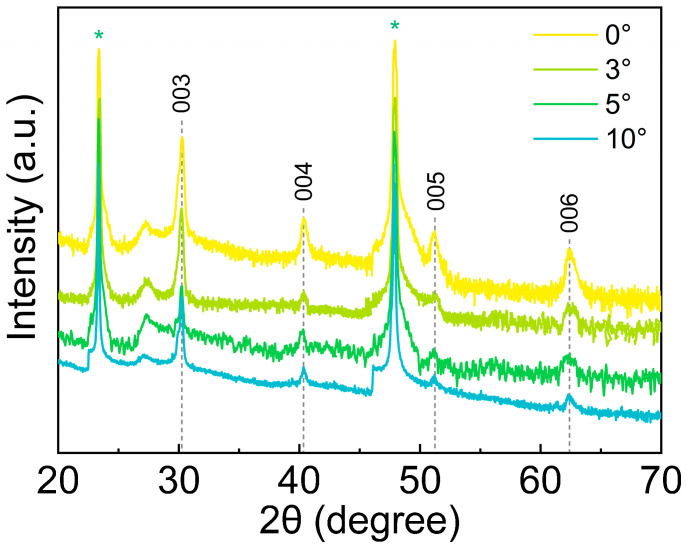
XRD *θ*-2*θ* scans of the Bi_0.94_La_0.06_CuSeO thin films with varying inclination angles (* denotes diffraction peaks of LaAlO_3_ substrates).

When the offset angles matched the tilted angles of the LaAlO_3_ substrate in an “Offset-Coupled” mode, only the (00*l*) diffraction peaks of the Bi_0.94_La_0.06_CuSeO thin films and the LaAlO_3_ substrates were observed from the XRD *θ*-2*θ* scans, as shown in Figure 2, which can be confirmed by the *PDF*#46-0296 of BiCuSeO. This indicates the growth of high-quality and pure-phase Bi_0.94_La_0.06_CuSeO thin films whose inclination angles consist with that of the applied substrates.

**Figure 3:**
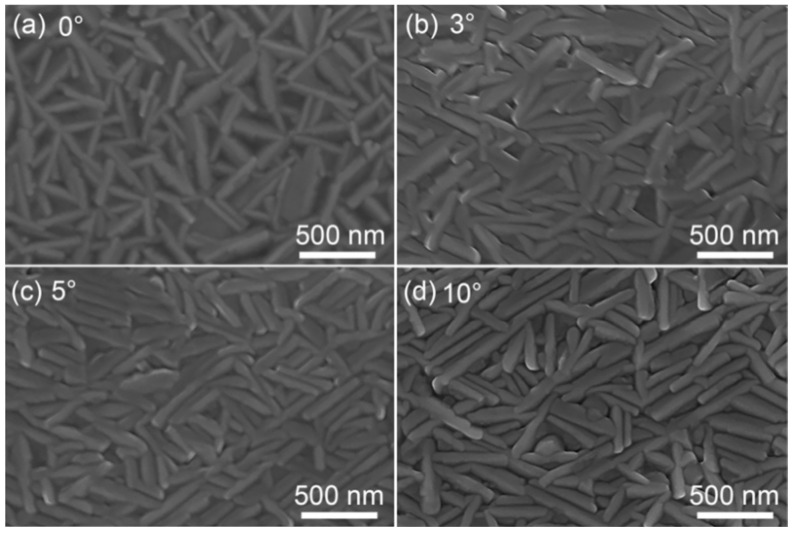
SEM images of the Bi_0.94_La_0.06_CuSeO thin films grown on (**a**) 0°-, (**b**) 3°-, (**c**) 5°- and (**d**) 10°-tilted LaAlO_3_ substrates.

The surface morphology of the films, characterized by SEM, is presented in Figure 3. All films exhibited rod-shaped grain particles with well-defined grain boundaries and no significant cracks or voids. As the inclination angle increased, the grain size became more uniform, and the film grew denser. Specifically, the width of rod-shaped grains was about 90 nm in non-inclined Bi_0.94_La_0.06_CuSeO thin film, whereas the width was only 63 nm in the 10° inclined Bi_0.94_La_0.06_CuSeO thin film.

### 3.2. Pulsed Photodetection

**Figure 4:**
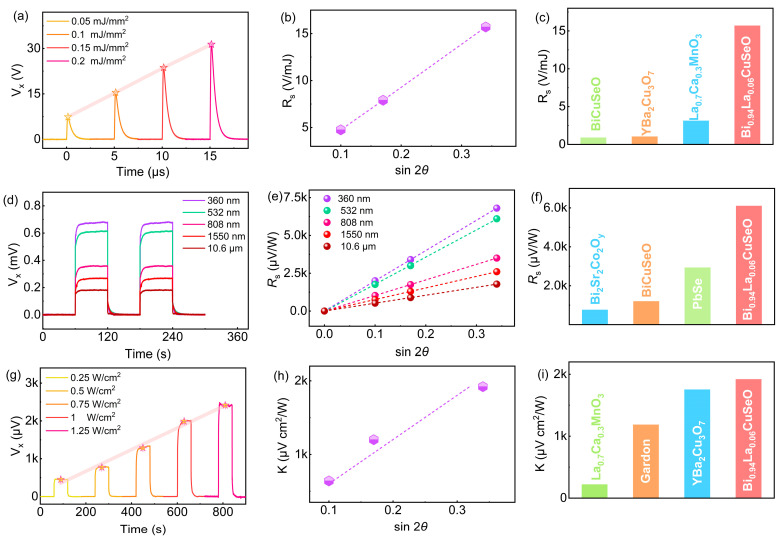
Voltage signals of inclined Bi_0.94_La_0.06_CuSeO thin film under different (**a**) energy densities, (**d**) power densities and (**g**) heat flux densities. (**b**,**e**,**h**) The relationship between sensitivity and sin2*θ* of inclined Bi_0.94_La_0.06_CuSeO thin film under different irradiations. (**c**,**f**,**i**) Comparison of sensitivity obtained in inclined Bi_0.94_La_0.06_CuSeO thin film with other materials and commercial Gardon gauge.

Figure 4a presents the voltage waveforms of the 10° inclined Bi_0.94_La_0.06_CuSeO thin film under 308 nm UV pulsed laser irradiation. The peak voltage (*V*_p_) exhibited a linear increase with *E*_d_, reaching a maximum of 31.4 V at 0.2 mJ/mm^2^. This linear relationship is crucial for practical photodetection applications. The considerably huge *V*_p_ may be attributed to the carrier concentration of 1.61 × 10^20^ cm^−3^, as reported in ref. [18]. In addition, the rise time (*τ*_r_), defined as the time of the voltage from 0 to *V*_p_, was measured to be only 148 ns, which was much faster than that of a commercial laser detector (8.8 ms, IP-550, Physcience Optoelectronic, Beijing, China). Such a fast response speed may due to the lower room-temperature resistivity of Bi_0.94_La_0.06_CuSeO thin film (about 275 μΩ m). It has been reported that the rise time increases monotonically with the penetration depth [6]. According to Equation (2), a lower resistivity will lead to a smaller optical penetration depth.

(2) $$\delta =\frac{\varepsilon_{0}\lambda c\rho }{4\pi }$$

where *ε*_0_ is the vacuum dielectric constant, *λ* is the incident light wavelength, *c* is the light speed, and *ρ* is the resistivity.

The sensitivity *R*_s_ (*R*_s_ = *V*_p_/*E*) varied linearly with sin2*θ*, peaking at 10°, as shown in Figure 4b. This behavior aligns well with the theoretical prediction in Equation (1), confirming that the voltage originates from the TTE effect due to the anisotropic Seebeck coefficient. A comparison of *R*_s_ values with other materials (Figure 4c) highlights the sensitivity of the inclined Bi_0.94_La_0.06_CuSeO thin film, which surpasses that of intrinsic BiCuSeO (0.9 V/mJ), YBa_2_Cu_3_O_7_ (1.04 V/mJ), and La_0.7_Ca_0.3_MnO_3_ (3.13 V/mJ) thin films [14,17,21]. These results stress the potential of the angle-dependent TTE effect in an inclined Bi_0.94_La_0.06_CuSeO thin film for weak UV pulsed laser detection.

### 3.3. CW Photodetection

In order to further investigate TTE detection performance under the irradiation of CW lasers, Figure 4d demonstrates the output voltage signals under CW laser irradiation across various wavelengths ranging from UV to FIR. Distinct voltage signals were observed without external bias or built-in electric field, exhibiting excellent switching characteristics as the laser was turned on or off. The output voltage amplitude increased linearly with the laser power density (*P*_d_, defined as the ratio of laser power *P* to irradiation area). Notably, the output voltage amplitude increased with the decreasing wavelength, reaching 0.68 mV under the irradiation of 360 nm CW laser at 9 W/cm^2^. The wavelength-dependent output voltage can be attributed to the light absorption coefficient (*γ*), which decreased as the irradiation wavelength increased. The strongest light absorption was obtained at 360 nm. According to the following one-dimensional thermal diffusion equation:

(3) $$∆T_{z}=\frac{P_{d}}{\gamma \rho CD}(\gamma d+e^{-\gamma d}-1)$$

where *P*_d_ is the power density of the CW laser, *κ* is the thermal conductivity, and *d* is the film thickness, a higher absorption coefficient results in a higher Δ*T* and thus a higher voltage sensitivity (*R*_s_, *R*_s_ = *V*_p_/*P*). The voltage sensitivity linearly increased with sin2*θ*, as illustrated in Figure 4e, peaking at 10°, a distinctive characteristic of the TTE effect that differentiates it from other photoelectric effects. The maximum *R*_s_ of 6100 μV/W at 10° exceeded that of the inclined Bi_2_Sr_2_Co_2_O_y_ (760 μV/W), BiCuSeO (1200 μV/W), and PbSe (2930 μV/W) thin films (Figure 4f) [3,17,22]. Additionally, the nonlinearity of the inclined Bi_0.94_La_0.06_CuSeO thin film was minimal (±0.97%), highlighting its suitability for high-precision sensor applications.

### 3.4. Heat Flux Detection

In addition, excellent thermal detection capability was also crucial for the film’s TTE performance. Significant output voltages along the 10° inclined Bi_0.94_La_0.06_CuSeO film were also detected under a point-like thermal source irradiation, as shown in Figure 4g. The voltage increased rapidly and stabilized as the heat source illuminated the film centre, indicating the establishment of a stable temperature difference along the thickness direction. The voltage increased linearly with heat flux density (*q*), reaching 2.4 mV at 1.25 W/cm^2^. The heat flux sensitivity (*K*, *K* = *V*_p_/*q*) peaked at approximately 1920 μV cm^2^/W for the 10° inclined Bi_0.94_La_0.06_CuSeO thin film, as shown in Figure 4h. The value was an order of magnitude higher than that of the La_0.7_Ca_0.3_MnO_3_ (220.9 μV cm^2^/W), twice that of the Gardon gauge (1186.7 μV cm^2^/W), and had an obvious advantage over YBa_2_Cu_3_O_7_ (1754 μV cm^2^/W) (Figure 4i) [13,16]. The high linearity (*R*^2^ = 0.9966) further confirms the potential of Bi_0.94_La_0.06_CuSeO thin film for designing high-performance TTE sensors. Our study offers an effective strategy for optimizing the detection sensitivity by controlling the inclination angle. The inclined Bi_0.94_La_0.06_CuSeO thin films exhibit excellent voltage sensitivity, linearity, and response characteristics, which are promising for advanced photodetection and thermal sensing applications.

## 4. Conclusions

In conclusion, the study systematically investigated the influence of the inclination angle on the photo and thermal detection performance of the Bi_0.94_La_0.06_CuSeO thin films. The inclined films demonstrated significant voltage generation under various irradiation sources without external bias voltage. A distinct linear relationship was observed between the output voltage and sin2*θ*. The 10° inclined Bi_0.94_La_0.06_CuSeO thin film achieved huge output voltages of 31.4 V, 0.68 mV, and 2.4 mV under the 308 nm pulsed laser, the 360 nm CW laser, and a point-like heat source irradiation, respectively. Notably, all responses exhibited excellent linearity, emphasizing the material’s reliability and precision for advanced detection applications. These findings highlight the potential of inclined Bi_0.94_La_0.06_CuSeO thin films as high-performance sensors for a wide range of TTE photothermal detection applications, confirming inclination angle optimization as an effective strategy for TTE performance enhancement.

## Data Availability

Data underlying the results presented in this paper are not publicly available at this time but may be obtained from the authors upon reasonable request.
